# Salmonella Bacteremia in an Older Patient With No Specific Entry: A Case Report

**DOI:** 10.7759/cureus.49194

**Published:** 2023-11-21

**Authors:** Koki Kudo, Junya Ohara, Chiaki Sano, Ryuichi Ohta

**Affiliations:** 1 Family Medicine, International University of Health and Welfare Graduate School of Health Sciences, Tokyo, JPN; 2 Family Medicine, Matsue Seikyo Hospital, Matsue, JPN; 3 Community Medicine Management, Shimane University Faculty of Medicine, Izumo, JPN; 4 Community Care, Unnan City Hospital, Unnan, JPN

**Keywords:** gastrointestinal tract, urinary tract infection, family medicine, general medicine, rural hospital, older, bacteremia, salmonella

## Abstract

In this case report, we describe a rare case of non-typhoidal *Salmonella* bacteremia in an 87-year-old woman with no apparent history of daily *Salmonella* exposure. The patient presented with fever, body discomfort, and diarrhea. Medical examinations ruled out usual sources of *Salmonella*, including raw food consumption and pet contact. Her medical history included postoperative sigmoid colon cancer, left breast cancer, and other ailments. Although *Salmonella* infection typically stems from oral intake, this case suggests that bacterial translocation from the gastrointestinal tract could be the primary cause, potentially exacerbated by the patient's age and medical history. Another hypothesis is an ascending infection from diarrhea to the urinary tract, which might have led to pyelonephritis and subsequent bacteremia. This case highlights the importance of recognizing the potential for severe infections such as sepsis in older individuals presenting with diverse symptoms. Therefore, this case further underscores the need for heightened clinical vigilance, especially in community hospitals, to ensure timely and appropriate management of such severe conditions in the older population.

## Introduction

*Salmonella* is a gram-negative, facultatively anaerobic, rod-shaped bacterium belonging to the *Enterobacteriaceae* family. It is an enterobacterium that lives mainly in the digestive tracts of humans and animals [1]. *Salmonella* is classified into two groups: those that cause typhoid fever and paratyphoid fever (*Salmonella* Typhi and *Salmonella* Paratyphi A), which are classified as class III infectious diseases, and non-typhoidal *Salmonella* (NTS), which cause infectious food poisoning [2]. Typhimurium is a serotype of NTS, and its official scientific name is *Salmonella enterica* subsp. *enterica* serovar Typhimurium [3]. The former is a systemic infection that causes bacteremia, whereas the latter mainly causes infectious enteritis and food poisoning and rarely causes bacteremia [4]. Conservative treatment is often selected, and the disease rarely becomes severe.

However, *Salmonella* infections in the blood can be severe. The entry of *Salmonella* during infections varies widely, and the immune status of older individuals may be a factor [5]. The primary route of *Salmonella* infection is oral [6]. It is generally believed that an average of approximately 106-109 or more organisms is required for disease onset [6]. However, according to a survey on actual outbreaks, the number of bacteria required for disease onset was calculated to be approximately 10-104 [3]. Symptoms of *Salmonella* infection include fever, headache, abdominal pain, diarrhea, and vomiting [6]. In healthy adults, symptoms are limited to gastroenteritis; however, in children and older individuals, the disease can become severe and can result in death from sepsis [7]. In this case report, we describe a case of NTS bacteremia in an older patient. There were no history of consumption of raw eggs or raw meat and no history of pet ownership. We believe that this case provides essential insights into the treatment of future cases of *Salmonella* bacteremia in older individuals and search for new entries.

## Case presentation

An 87-year-old woman visited the emergency department of a rural community hospital with a chief complaint of fever and difficulty moving her body. Until the day before admission, she was able to lead her daily life without any significant changes. On the morning of admission, she developed a fever of 38°C and diarrhea at a day service. She had not consumed any food that might pose a risk of infection until one week prior and had not consumed raw chicken eggs, raw meat, eels, or soft-shelled turtles. She also did not keep pets or reptiles such as green turtles and had no international travel history. She had a history of postoperative sigmoid colon cancer, postoperative left breast cancer, hypothyroidism, type 2 diabetes mellitus, hypertension, Alzheimer's disease, and a humeral fracture. Her medical history included administration of donepezil (5 mg).

The vital signs at the visit were as follows: blood pressure, 136/80 mmHg; pulse rate, 96 beats/min; body temperature, 37.8°C; respiratory rate, 22 breaths/min; and oxygen saturation, 96% on room air. The patient was alert to time, place, and person. A physical examination revealed no lymphadenopathy. No abnormal heart or respiratory sounds were observed. The abdomen was flat and soft, with no spontaneous pain, tenderness over the entire abdomen, or hyperintestinal peristaltic sounds. There were no costovertebral angle tenderness. No edema or skin rash was observed on the extremities. Blood counts showed a normal white blood cell count but a predominance of neutrophils. Biochemical examinations revealed elevated C-reactive protein levels (Table 1).

**Table 1:** Initial laboratory test data of the patient eGFR: estimated glomerular filtration rate; Na: sodium; K: potassium; Cl: chlorine

Parameter	Value	Reference
White blood cell count	8.20	3.5-9.1 × 10^3^/μL
Neutrophil differential count	90.5%	44.0-72.0%
Lymphocyte differential count	7.1%	18.0-59.0%
Monocyte differential count	2.0%	0.0-12.0%
Eosinophil differential count	0.0%	0.0-10.0%
Basophil differential count	0.4%	0.0-3.0%
Red blood cell count	4.04	3.76-5.50 × 10^6^/μL
Hemoglobin level	11.0	11.3-15.2 g/dL
Hematocrit volume	33.5%	33.4-44.9%
Mean corpuscular volume	82.9	79.0-100.0 fl
Platelet count	15.8	13.0-36.9 × 10^4^/μL
Total protein level	6.0	6.5-8.3 g/dL
Albumin level	3.7	3.8-5.3 g/dL
Total bilirubin level	0.9	0.2-1.2 mg/dL
Aspartate aminotransferase level	35	8-38 IU/L
Alanine aminotransferase level	18	4-43 IU/L
Alkaline phosphatase level	96	106-322 U/L
γ-Glutamyl transpeptidase level	20	<48 IU/L
Lactate dehydrogenase level	287	121-245 U/L
Blood urea nitrogen level	17.2	8-20 mg/dL
Creatinine level	0.60	0.40-1.10 mg/dL
eGFR	69.5	>60.0 mL/min/L
Serum Na level	137	135-150 mEq/L
Serum K level	3.5	3.5-5.3 mEq/L
Serum Cl level	100	98-110 mEq/L
Creatine kinase level	104	56-244 U/L
C-reactive protein level	7.21	<0.30 mg/dL

A simple computed tomography (CT) scan of the abdomen revealed no fluid accumulation in the small or large intestines. Two sets of blood and urine cultures were performed upon admission.

**Figure 1:**
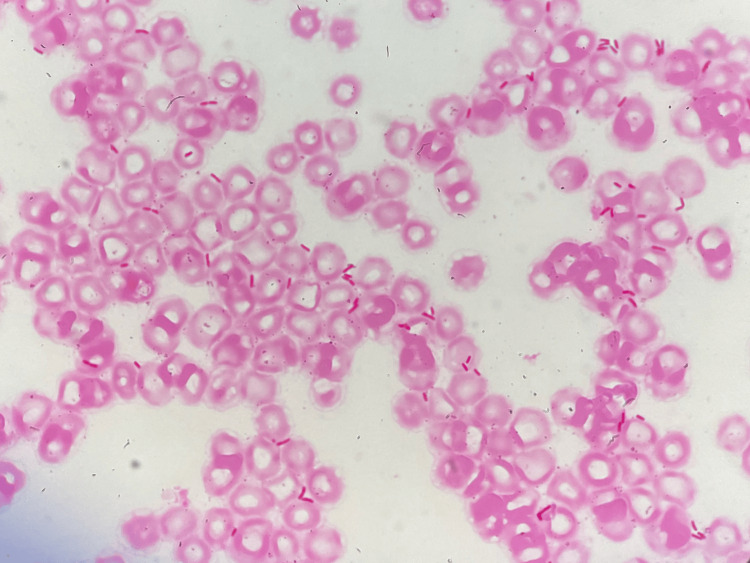
Gram stain of blood culture showing gram-negative rods dispersed throughout the field (×400)

On the first day of admission, the fever increased to 39.1°C. Based on diarrheal symptoms and imaging findings, infectious enteritis was diagnosed, and the patient was treated with fluid replacement and symptomatic therapy. On the second day, a blood culture revealed the presence of gram-negative bacilli (Figure 1).

Cefmetazole (4 g/day) was administered, considering infectious enteritis led to bacteremia. On the seventh day, *Salmonella* sp. was identified in two sets of blood, urine, and stool cultures. Drug susceptibility testing showed that the patient was sensitive to ampicillin at a dose of 4 g/day, and a change in administration was made. On the seventh day, cardiac ultrasonography was performed for a detailed examination of infective endocarditis caused by *Salmonella* bacteremia without any abnormalities. Contrast-enhanced CT was performed on the eighth day, investigating any inflammation and abscess formations for the detection of entry of the bacteria. However, no abscess formation or apparent lesions were observed in the skull, lungs, pleura, liver, kidneys, iliopsoas muscle, or large blood vessels. The patient's abdominal pain improved after the second day of illness, and diarrhea persisted until the ninth day. Thereafter, there was no diarrhea or abdominal pain, and the patient recovered to the point where she could consume all her food. On the 29th day, a fecal occult blood test was performed as part of searching for colon cancer; however, the results were negative. On the 31st day, the patient was transferred to a rehabilitation ward and discharged.

## Discussion

In this case report, we present the case of an older patient with *Salmonella* bacteremia of unknown etiology. *Salmonella* bacteremia in older patients with no history of *Salmonella* exposure is rare, and its entry is most likely due to contact infection or bacterial translocation from endemic *Salmonella* in the intestinal tract [7]. However, there have been no reports of its progression to *Salmonella* bacteremia. Our case highlights the importance of considering the possibility of *Salmonella* colonization in the intestinal tract of older patients and the possibility of bacterial translocation, including *Salmonella*, in older patients who become severely ill due to diarrheal symptoms.

Bacterial translocation from the gastrointestinal tract should be considered first as an entry point for *Salmonella* bacteremia, which includes typhoidal salmonellosis caused by *Salmonella* Typhi and *Salmonella* Paratyphi A and non-typhoidal salmonellosis caused by other species of bacteria. Salmonellosis can be classified into typhoidal *Salmonella*, caused by *Salmonella* Typhi and *Salmonella* Paratyphi A, and NTS, caused by other species [4,8]. NTS is found in the intestinal tract of domestic animals such as chickens, pigs, and cows, as well as in reptiles and rodents [9]. NTS bacteremia is transmitted by the oral ingestion of *Salmonella* entering the intestinal tract, where macrophages phagocytose them, and they reach the mesenteric lymph nodes via macrophages to avoid bactericidal mechanisms, leading to proliferation in the lymph nodes and bacteremia [10].

In addition, older individuals are at a risk of developing NTS bacteremia. The patient had a history of postoperative sigmoid colon and left breast cancer, and that she was of older age and had a malignant tumor were predisposing factors. Although there have been reports of *Salmonella* bacteremia in patients with cancer, there have been few reports of *Salmonella* bacteremia in older patients in Japan, and this novel case suggests the possibility of such cases [11,12]. No infection sites were observed in this case, and *Salmonella* was detected in the blood, urine, and stool. The main factors that cause bacterial translocation include changes in the intestinal microbiota, weakening of the intestinal mucosa, and host immunity [13]. The modes of occurrence have been reported to include direct breakthroughs of the intestinal epithelium, intercellular breakthroughs, and phagocytosis by macrophages [6]. In the present case, changes in the intestinal microbiota, intestinal immunity, fragile intestinal mucosa, and host immunity were all associated with the patient's advanced age and postoperative sigmoid colon cancer, and bacterial translocation was likely the cause of the disease [13].

Another possibility for *Salmonella* bacteremia is that *Salmonella* migration from the gastrointestinal tract to the urinary tract may have led to pyelonephritis and bacteremia [14]. In *Salmonella* infection, diarrhea is watery and sometimes contains mucous membranes or blood, which are quickly transmitted to the urinary tract [15]. In this case, the patient presented with fever, abdominal tenderness, and watery diarrhea in large quantities when brought to the emergency room. The details of when the diarrhea began may not be clear because of patient's age and cognitive functions, and we cannot rule out the possibility that the diarrhea preceded the onset of the disease before the patient's arrival. Gram staining of the urine also revealed gram-negative bacilli. Although urinalysis and physical examination findings were not suggestive of pyelonephritis, the urine and blood cultures were positive for *Salmonella*, suggesting that the patient may have had pyelonephritis and that diarrhea may have caused an ascending infection through the urethra, leading to a blood infection from pyelonephritis, which could have led to bacteremia. The patient's condition may have been caused by the diarrhea.

This case highlights the importance of suspecting sepsis and bacteremia in older patients presenting with various symptoms. We believe that medical professionals working in community hospitals, including general physicians, should always be aware of the possibility of fatal sepsis due to various symptoms and respond appropriately to these symptoms to improve the prognosis of older patients in community hospitals [16,17].

## Conclusions

In this case report, an 87-year-old woman with no apparent exposure to familiar sources of *Salmonella* developed NTS bacteremia. Despite having no known risk factors, such as consumption of raw eggs or raw meat or contact with pets, she exhibited severe symptoms, prompting a search for the source of infection. The report concluded that bacterial translocation from the gastrointestinal tract, possibly exacerbated by the patient's age and medical history, may have been a key factor in the bacteremia, underscoring the need for clinicians in community hospitals to be vigilant of signs of sepsis in older patients with diverse symptoms.
